# Teachers’ Well-Being and Innovative Work Behavior: A Moderated Mediation Model of Perceived Insider Status and Principal Authentic Leadership

**DOI:** 10.3390/bs15101419

**Published:** 2025-10-19

**Authors:** Chao Lu, Zeqing Xu, Qinrui Tian

**Affiliations:** 1 Institute of Basic Education Development, Shanghai Normal University, Shanghai 200234, China; luchao@shnu.edu.cn (C.L.); 1000546583@smail.shnu.edu.cn (Q.T.); 2 Department of Education Management, Faculty of Education, East China Normal University, Shanghai 200062, China

**Keywords:** teacher well-being, innovative work behavior, perceived insider status, principal authentic leadership, moderated mediation

## Abstract

Teacher innovation is critical for fostering student creativity, enhancing school effectiveness, and advancing national talent strategies. Grounded in the broaden-and-build theory of positive emotions and social information processing theory, this study develops a moderated mediation model to explore the motivational mechanisms underlying teachers’ innovative work behavior. Using survey data from 508 teachers in mainland China, the analysis reveals that teacher well-being positively influences innovative work behavior, and this relationship is mediated by perceived insider status. Furthermore, principal authentic leadership enhances the impact of perceived insider status on innovation and strengthens the indirect effect of well-being through this mediator. These findings underscore the importance of both emotional pathways and contextual signals in shaping teacher innovation, offering theoretical contributions to education leadership and teacher work behavior research while providing practical implications for creating supportive and innovation-conducive school environments.

## 1. Introduction

The rapid advancement of artificial intelligence, global competition, and curriculum reforms have introduced unprecedented complexity and uncertainty into basic education (Chen et al., 2025). Teachers, as central actors who translate policy into classroom practice, are pivotal in cultivating student creativity, enhancing school effectiveness, and supporting national talent strategies (Pietsch et al., 2024; Fullan, 2009). In China, however, teacher innovation often encounters a paradox: while external demands for accountability and reform intensify, teachers’ intrinsic motivation to innovate remains limited. Substantial investments in digital infrastructure and curriculum restructuring have not always translated into meaningful innovation (L. Zhang & Liu, 2019), as teachers face rising stress, workload pressures, and professional burnout that constrain their capacity to respond proactively to reform demands (Zhao et al., 2022). Consequently, understanding the drivers of teacher innovation has become a pressing agenda in educational research.

Previous studies have identified various antecedents of teacher innovation, including individual traits (e.g., self-efficacy), organizational factors (e.g., leadership), and institutional context (e.g., policy alignment) (Thurlings et al., 2015; B. Wu et al., 2024). However, teachers often face the compounded pressures of administrative duties, instructional responsibilities, and parent–school engagement nowadays (Avola et al., 2025). Under such conditions, well-being serves not only as an indicator of emotional state but also as a deeper reflection of professional commitment and perceived organizational support (Xu & Pang, 2024). Teachers with higher well-being may be more inclined to reframe reform-related pressures as opportunities for professional growth, thereby activating their intrinsic motivation to innovate. In contrast, findings from organizational research on employee innovation present a more nuanced picture. While some studies suggest that well-being positively predicts innovative behavior (J. Wang et al., 2017), others report a negative association between work-related happiness and innovation performance (J. Zhou & George, 2001). These inconsistencies highlight the need to investigate whether, and how, teacher well-being translates into innovative practices in school contexts.

To deepen this understanding, the present study introduces two key variables. First, perceived insider status is proposed as a mediator. Influenced by Confucian values such as ‘guanxi’ and collectivism, Chinese teachers are particularly sensitive to whether they are treated as ‘insiders’ within the school community (Hwang, 2000). When teachers perceive a strong sense of insider identity, they are more likely to feel collective responsibility and be less concerned about the reputational risks of failure, thus increasing the likelihood that their well-being will translate into sustained innovation. Second, principal authentic leadership is chosen as a moderator because leadership serves as a key contextual factor shaping innovation (Scott & Bruce, 1994). Authentic leadership represents a genuine, hopeful, and visionary response to organizational challenges, characterized by openness, moral integrity, and creativity (W. Zhang et al., 2023). Under such leadership, teachers are more likely to experience greater well-being and receive the necessary support from leaders to implement innovative teaching practices. By examining its moderating role, this study responds to calls for research beyond predominantly Western contexts (Duignan, 2014), contributing to a more culturally inclusive understanding of how authentic leadership fosters teacher innovation in diverse educational settings.

Therefore, the research questions guiding this study are as follows: (1) Does teacher well-being directly predict innovative work behavior? (2) Does perceived insider status mediate this relationship? (3) How does principal authentic leadership moderate the pathway?

## 2. Literature Review and Hypotheses

### 2.1. Broaden-And-Build Theory of Positive Emotions and Social Information Processing Theory

The broaden-and-build theory of positive emotions, first proposed by Fredrickson (2001), posits that positive emotions expand individuals’ momentary thought–action repertoires and contribute to the development of enduring personal resources, ultimately enhancing long-term adaptability. This theoretical lens offers a valuable foundation for understanding how teacher well-being influences innovation. Specifically, when teachers experience higher levels of well-being, they tend to feel more frequent and intense positive emotions such as joy, gratitude, and contentment. These emotions broaden attention and foster cognitive flexibility (M. Li et al., 2024), increasing teachers’ sense of belonging and identification with the school—that is, their perceived insider status. Over time, positive emotions also help build enduring psychological resources such as creativity, resilience, and job performance (Kiken & Fredrickson, 2017). Thus, teacher well-being may indirectly promote innovative work behavior by enhancing perceived insider status.

Social Information Processing Theory further explains how contextual cues shape individual behavior (Gurbin, 2015). It posits that individuals interpret and respond to social information to guide their attitudes and actions (Salancik & Pfeffer, 1978). In school settings, principals serve as key sources of contextual cues that shape teachers’ perceptions and behaviors. As authentic leaders, they demonstrate positive psychological capacities and moral virtues, cultivating trust and mutual growth through transparent communication and consistent role modeling (Avolio & Gardner, 2005). When teachers perceive such leadership, they are more likely to internalize organizational values, develop a strong sense of being valued insiders, and translate their well-being into innovative practices. In contrast, inauthentic leadership may undermine trust, weaken organizational identification, and ultimately inhibit innovation.

**Figure 1:**
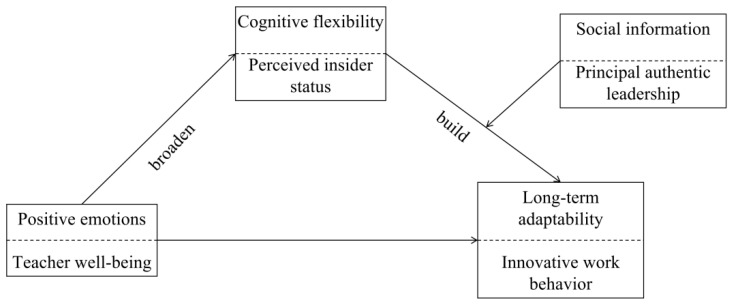
Theoretical framework.

Based on the above analysis, we propose a moderated mediation model shown in Figure 1.

### 2.2. Teachers’ Well-Being and Innovative Work Behavior

Teacher innovative work behavior (TIWB) refers to a set of proactive activities through which teachers continuously update their pedagogical beliefs and apply novel ideas in teaching practice to benefit students, enhance school performance, and foster personal development (Janssen, 2003). As innovation becomes central to educational transformation, research on TIWB has gained increasing attention. Existing studies primarily examine its antecedents from two perspectives: (1) individual attributes such as achievement motivation (Noviyanti et al., 2021), self-efficacy (X. Huang et al., 2019), and psychological need satisfaction (Messmann et al., 2022); and (2) organizational contexts, including principal leadership (Rais & Rubini, 2022), resource availability (Messmann & Mulder, 2011), and school climate (D. Wu et al., 2022). More recent work has begun to uncover the ‘black box’ mechanisms underlying teacher innovation by exploring the mediating roles of interpersonal interaction factors like collaboration (Ma & Zhang, 2025), knowledge sharing (Eva et al., 2020), and leader–member exchange (Vermeulen et al., 2022).

Teachers’ well-being (TWB) may serve as a critical internal driver of innovative work behavior. As a multidimensional psychological construct encompassing teachers’ affective states and cognitive appraisals (Viac & Fraser, 2020). Higher levels of TWB are associated with greater resilience, enabling teachers to manage stress effectively, recover from setbacks in instructional innovation, and maintain motivation despite challenges (Beltman et al., 2011); it also fosters stronger organizational commitment, encouraging teachers to proactively contribute innovative ideas for school improvement and actively share teaching practices with colleagues (W. Huang et al., 2023). Numerous studies have provided empirical evidence of the positive relationship between Teachers’ well-being and innovative work behavior. Messmann and Mulder (2011) found that teachers with higher job satisfaction were more willing to invest extra effort in implementing innovative teaching strategies. Similarly, Yu et al. (2007) found that job satisfaction is positively related to playfulness, an affective trait associated with creativity. In the Malaysian context, Johari et al. (2021) also showed that teachers’ experience of positive emotions (e.g., humor) at work significantly predicted their innovative behaviors.

Hence, we propose

**Hypothesis 1:**  *Teachers’ well-being positively predicts innovative work behavior.*

### 2.3. Teachers’ Perceived Insider Status as a Mediator

As a relational psychological resource, teacher well-being (TWB) not only mitigates burnout (Avola et al., 2025) but also enhances teachers’ sense of belonging and organizational identification (X. Li et al., 2024). High levels of TWB suggest that teachers’ contributions are recognized and valued by school leaders and colleagues (Tsuyuguchi, 2023). Consequently, teachers are more likely to attribute their sense of satisfaction and achievement to the supportive environment provided by the school, reinforcing their alignment with its goals and values and thus strengthening their perceived insider status (Tang et al., 2018). Perceived Insider Status (PIS) refers to the extent to which teachers perceive themselves as being accepted as ‘insiders’ within the school organization (Masterson & Stamper, 2003). Research in the field of organizational behavior has confirmed the positive association between well-being and PIS. For instance, Aldabbas and Bettayeb (2024), using cross-industry data from the United Arab Emirates, found that subjective well-being indirectly enhanced PIS by promoting employees’ positive interpretations of managerial care. When individuals experience heightened emotional vitality and psychological safety, they are more inclined to identify themselves as organizational insiders (Manville et al., 2023).

Based on the evidence, we propose

**Hypothesis 2a:**  *Teachers’ well-being is positively related to perceived insider status.*

Teachers with higher perceived insider status are more likely to engage in innovative work behavior. When teachers perceive themselves as genuine insiders, they experience greater psychological safety and work engagement (Schaubroeck et al., 2017; Mavrommatidou et al., 2023), which foster a willingness to identify challenges, generate novel ideas, and implement innovative teaching practices (Horng et al., 2016). Bao’s (2025) empirical findings further indicate that when teachers view themselves as indispensable to the organization, a reciprocal mechanism of social exchange is activated: teachers are motivated to exceed role expectations through innovative behaviors as a form of reciprocation, thereby reinforcing their internal status and self-worth. Moreover, within the collectivist context of Chinese organizational culture, teachers with a strong sense of group belonging tend to prioritize organizational over personal interests (S. Zhang et al., 2021). This orientation encourages them to proactively address organizational challenges and contribute to team well-being, fostering altruistic and innovative behaviors.

Hence, we propose

**Hypothesis 2b:**  *Perceived insider status is positively related to teachers’ innovative work behavior.*

As previously discussed, positive emotions broaden teachers’ cognitive and psychological resources, helping to build the foundation for innovation. However, perceived insider status acts as a gatekeeper, determining whether these emotional benefits lead to actual innovative efforts. Teachers are most likely to innovate when they feel both emotionally positive and strongly connected to their school. In contrast, even if teachers experience positive emotions, those who see themselves as outsiders may become less engaged and more emotionally exhausted (Hwang, 2001). This weakens their motivation to innovate, leaving their innovative potential unrealized.

Prior research has identified that PIS, as a manifestation of these psychological resources, serves as a key mechanism through which well-being translates into innovative behavior (J. Wang & Kim, 2013). Lee and Hyun (2016) found that PIS mediated the relationship between positive psychological experiences and service innovation among airline employees. More recent evidence further highlights the mediating role of PIS. Gigant et al. (2025) showed that team support, social cohesion, and intra-team trust enhance individuals’ sense of insider status and belonging, which in turn strengthens their engagement and innovative contributions. Likewise, Y. Li and Jia (2024), drawing on data from a large Chinese financial institution, revealed that PIS mediated the effects of leader–follower cognitive style congruence on employees’ psychological distress, underscoring its broader role in shaping both well-being and behavioral outcomes.

Accordingly, we propose

**Hypothesis 3:**  *Perceived insider status mediates the relationship between teachers’ well-being and innovative work behavior.*

### 2.4. Principal Authentic Leadership as a Moderator

According to social information processing theory, leaders serve as key sources of interpretive cues in complex and ambiguous work environments. In schools, where formal authority structures are strong and teacher autonomy is often constrained, principal leadership becomes particularly salient in shaping teachers’ perceptions of organizational inclusion and support (Gardner et al., 2005). Authentic leadership, defined as ‘a process that draws from both positive psychological capacities and a highly developed organizational context, which results in both greater self-awareness and self-regulated positive behaviours on the part of leaders and associates, fostering positive self-development’ (Luthans & Avolio, 2003), provides a critical signal in this process.

When teachers perceive their principals as authentic, they are more likely to interpret organizational messages as trustworthy and inclusive, thereby reinforcing their perceived insider status (Grandey et al., 2012). Principals high in self-awareness understand their values and emotions, fostering reflective practices and psychological safety that support innovation. Relational transparency involves open and genuine communication, which builds trust and reduces teachers’ perceived risks in trying new approaches. An internalized moral perspective grounds leadership in ethical values and collective purpose, motivating teachers to contribute to organizational improvement beyond self-interest. Balanced processing enables principals to consider diverse viewpoints objectively, promoting an inclusive environment for critical and creative thinking (Avolio et al., 2004). To sum up, these characteristics of authentic leadership enhance teachers’ psychological empowerment and organizational identification, thereby strengthening their motivation and capacity for innovative work behavior (Grošelj et al., 2021). Xiong et al. (2016) further demonstrated that employees under authentic leaders are more inclined to internalize organizational goals and contribute beyond their formal roles.

Based on the above analysis, we suppose the following.

**Hypothesis 4:**  *Principals’ authentic leadership positively moderates the relationship between perceived insider status and teacher innovative work behavior.*

Furthermore, by combining Hypothesis 3 and Hypothesis 4, it is plausible that authentic leadership also moderates the strength of the indirect effect of teacher well-being on innovation through perceived insider status. In schools with high levels of authentic leadership, the climate of trust and psychological safety enables teachers to transform their well-being into stronger identification with the school community (Kiersch & Byrne, 2015). This enhanced insider perception motivates teachers to reciprocate with discretionary and innovative efforts (Kılınç et al., 2022). In contrast, schools with weak authentic leadership often feature opaque decision-making and inconsistent moral signaling (Fox et al., 2015). In such environments, teachers may doubt their organizational role and become less willing to engage in innovation, even if they have high levels of well-being. This conditional indirect effect aligns with findings by H. U. I. Wang et al. (2014), who showed that authentic leadership enhances the influence of positive psychological states on workplace behavior. It also echoes the argument by Zubair and Kamal (2015) that authentic leaders serve as amplifiers of employee creativity by shaping emotional and cognitive frames.

Therefore, we suppose the following.

**Hypothesis 5:**  *Principal authentic leadership moderates the mediating effect of perceived insider status on the relationship between teachers’ well-being and innovative work behavior, such that the indirect effect is stronger when authentic leadership is high.*

## 3. Research Methodology

### 3.1. Data Collection and Participants

This study was conducted in City B, recognized in China for its high-quality educational resources and skilled teaching workforce. To ensure sample diversity and representativeness, stratified cluster sampling method was employed. The schools in City B were categorized into strata based on two criteria: (a) geographic location (urban vs. suburban districts) and (b) school development level (high-performing vs. average-performing, according to local education bureau reports). Within each stratum, schools were randomly selected to form the sample, resulting in nine public primary and secondary schools. Each selected school represented a cluster, and all full-time teachers within these clusters were invited to participate. Data were collected via paper-based questionnaires administered on-site by trained graduate students majoring in education. To ensure procedural rigor and minimize social desirability bias, researchers provided standardized instructions and emphasized the voluntary nature of participation, along with anonymity and confidentiality. No personal identifiers were collected, and all participants provided written informed consent.

A total of 616 questionnaires were distributed and returned immediately after completion. After removing incomplete or invalid responses, 508 valid questionnaires were retained for analysis, yielding a valid response rate of 82.47%. Among the valid respondents, 81.30% were female and 18.70% male, which reflects the gender composition typical of Chinese primary and secondary schools. In terms of educational background, 87.80% held a bachelor’s degree, 8.66% a master’s degree, and 3.54% a junior college diploma. Regarding teaching experience, 31.69% of teachers had worked for more than 26 years, followed by 21.06% with 21–25 years of service. In terms of professional roles, 81.30% were front-line classroom teachers, 17.32% held mid-level leadership positions or above, and 1.38% were staff engaged in administrative, financial, or logistical support roles.

### 3.2. Measures

All key constructs in this study were assessed using validated scales that have been adapted to the Chinese educational context. Confirmatory factor analyses (CFA) were conducted for each scale to verify their construct validity. Internal consistency was assessed using Cronbach’s alpha coefficients. As summarized in Table 1, all scales showed acceptable reliability and validity.

#### 3.2.1. Teachers’ Well-Being

Teachers’ well-being was measured using the scale originally developed by Zheng et al. (2015), which consists of three subdimensions: life well-being (6 items), work well-being (6 items), and psychological well-being (6 items). Sample items include “Overall, I am very satisfied with the work I do” and “My job is very interesting.” Respondents rated each item on a 7-point Likert scale ranging from 1 (strongly disagree) to 7 (strongly agree), with higher scores indicating a greater level of well-being. The scale demonstrated excellent internal consistency (Cronbach’s α = 0.961). CFA results showed a good model fit: χ^2^/df = 5.498, RMSEA = 0.094, SRMR = 0.040, CFI = 0.936, NFI = 0.923.

#### 3.2.2. Teachers’ Innovative Work Behavior

Teachers’ innovative work behavior was assessed using a revised version of the scale developed by Janssen (2000) and adapted by M. Li (2016) for Chinese primary and secondary school teachers. The scale comprises two dimensions: generation of innovative ideas (4 items) and implementation of innovative practices (7 items). Items were rated on a 5-point Likert scale from 1 (never) to 5 (always). Sample items include “I often come up with new ideas to support student development” and “I frequently apply new ideas in my teaching practice.” The scale showed high internal reliability (Cronbach’s α = 0.973), with CFA indicating good model fit: χ^2^/df = 5.515, RMSEA = 0.094, SRMR = 0.022, CFI = 0.976, NFI = 0.971.

#### 3.2.3. Perceived Insider Status

Perceived insider status was measured using the scale developed by Stamper and Masterson (2002). A translation and back-translation procedure was used to ensure cross-cultural validity, and minor wording adjustments were made to suit the Chinese educational context (Mao et al., 2024). Four items were rated on a 5-point Likert scale (1 = strongly disagree, 5 = strongly agree). A representative item is “I strongly feel that I am a part of this school.” The scale demonstrated good reliability (Cronbach’s α = 0.860), with satisfactory CFA results: χ^2^/df = 4.093, RMSEA = 0.078, SRMR = 0.023, CFI = 0.995, NFI = 0.994.

#### 3.2.4. Authentic Leadership

Authentic leadership was measured using the scale developed by Walumbwa et al. (2008) and revised by S. Zhang and Mao (2020). In line with prior research, authentic leadership in this study refers to teachers’ perceptions of their principals’ authentic leadership behaviors, rather than principals’ self-reported leadership style. The scale consists of four dimensions: relational transparency (5 items), internalized moral perspective (3 items), balanced processing (3 items), and self-awareness (4 items). Responses were given on a 5-point Likert scale. Higher scores indicate teachers’ stronger perceptions of the principal authentic leadership. Sample items include “The principal encourages others to voice their opinions, even if they challenge existing views,” and “The principal clearly expresses his or her ideas.” The scale demonstrated excellent reliability (Cronbach’s α = 0.966), and CFA results supported its validity: χ^2^/df = 5.835, RMSEA = 0.098, SRMR = 0.030, CFI = 0.961, NFI = 0.954.

#### 3.2.5. Control Variables

Based on previous research showing that certain teacher characteristics are related to innovative work behavior, this study controlled for gender, age, years of teaching experience, educational background, homeroom teacher status, and job position. Among them, gender, homeroom teacher status, and job position were treated as categorical variables and were dummy coded. For gender, female and male were included, with male as the reference group. For homeroom teacher status, the categories were yes and no, with no as the reference group. For job position, three groups were defined: regular teachers, middle-level and senior leaders (including subject leaders, grade coordinators, academic and moral affairs directors, and vice principals), and other staff (such as finance and administrative personnel), with the last group serving as the reference group.

### 3.3. Data Analysis Strategy

All analyses were executed using SPSS 20.0 and AMOS 21.0. Confirmatory factor analysis (CFA) was used to assess the reliability and validity of all measurement scales. Common method bias was evaluated through both procedural safeguards and statistical checks. Descriptive statistics and inter-variable correlations were also computed. Multiple linear regression analyses were performed to examine direct effects. The mediating role of perceived insider status was tested using the PROCESS macro (Model 4) with 5000 bootstrap resamples. For the moderated mediation model, PROCESS Model 14 was applied.

### 3.4. Preliminary Data Analysis

Several preliminary tests were conducted prior to hypothesis testing. First, the issue of common method biases (CMBs) often arises when data are collected from a single source using self-report questionnaires. To mitigate this, we implemented several procedural remedies during the data collection process, such as ensuring participant anonymity, using clear and neutral wording with reverse-coded items, and randomizing item order. Additionally, following the recommendations of H. Zhou and Long (2004), a confirmatory factor analysis (CFA) was conducted in which all measurement items were loaded onto a single latent factor. The results indicated poor model fit (χ^2^/df = 12.914, RMSEA = 0.153, SRMR = 0.168, CFI = 0.566, NFI = 0.547), rejecting the hypothesis that a single factor could account for the majority of variance. This suggests that common method bias is not a serious concern in this study (Podsakoff et al., 2003).

Second, discriminant validity among the key constructs was assessed using CFA. Table 2 presents the results of measurement models. The proposed four-factor model exhibited significantly better fit than alternative models (χ^2^/df = 3.747, RMSEA = 0.074, RMR = 0.038, CFI = 0.900, NFI = 0.869), indicating that the four variables are empirically distinct and demonstrate satisfactory discriminant validity.

Finally, descriptive statistics and Pearson correlation coefficients were calculated using SPSS 20.0. As shown in Table 3, all key variables were positively and significantly correlated, providing initial support for the hypothesized relationships among teachers’ well-being, perceived insider status, innovative work behavior, and principal authentic leadership.

## 4. Results

### 4.1. Main Effects and Moderating Effects

Multiple linear regression using ordinary least squares was employed to test both main and moderating effects. When conducting hierarchical regression analyses, we entered gender, homeroom teacher status, position, age, education, and teaching experience as control variables. All predictors were mean-centered prior to creating interaction terms. Table 4 summarizes the results of the regression analysis.

**Figure 2:**
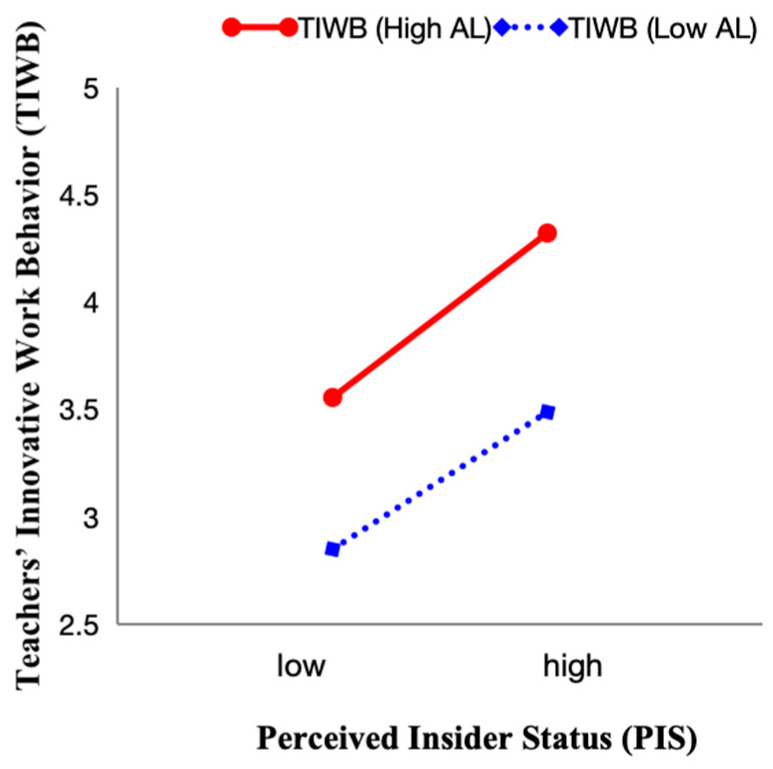
Moderating effect of authentic leadership on perceived insider status and teachers’ innovative work behavior.

Model 2 regressed teacher innovative work behavior on well-being and found a significant positive effect (β = 0.483, *p* < 0.01), supporting H1. Beyond statistical significance, the explanatory power of the model also increased substantially, with R^2^ rising from 0.032 to 0.310. This indicates that teacher well-being explained 27.8% of the variance in innovative work behavior, highlighting its substantive contribution. Model 4 showed that teacher well-being positively predicted perceived insider status (β = 0.466, *p* < 0.01), supporting H2a. The explained variance increased by 21.9%, underscoring the role of teacher well-being as a strong predictor of perceived insider status. Model 6 confirmed that perceived insider status significantly predicted innovative behavior (β = 0.560, *p* < 0.01), supporting H2b. The R^2^ value rose from 0.032 to 0.277, showing that perceived insider status accounted for 24.5% of the variance in innovative work behavior. In Model 8, the interaction between perceived insider status and authentic leadership was significant (β = 0.243, *p* = 0.002 < 0.01), supporting H4. As illustrated in Figure 2, the positive relationship between insider status and innovation was stronger when principal authentic leadership was high.

### 4.2. Mediation Analysis

To test the mediating role of perceived insider status, we followed the procedure developed by Preacher et al. (2007), using Model 4 in the PROCESS macro. A bootstrap resampling method with 5000 iterations and 95% confidence intervals was applied. If the confidence interval for the indirect effect does not include zero, the mediation is considered significant. The results in Table 5 show that the indirect effect of teachers’ well-being on innovative work behavior through perceived insider status was significant (indirect effect = 0.151, *p* < 0.01, 95% Bias-Corrected CI = [0.108, 0.227]). The indirect pathway accounted for 31.30% of the total effect, confirming Hypothesis 3.

### 4.3. Moderated Mediation Analysis

To examine whether the indirect effect of teachers’ well-being on innovative work behavior through perceived insider status varies depending on the level of authentic leadership, we conducted an analysis using Model 14 of the PROCESS macro, following the guidelines of Muller et al. (2005). A bootstrapping procedure with 5000 resamples and a 95% confidence level was used to ensure the robustness of the results. As shown in Table 6, when authentic leadership was low (Z = 3.984), the 95% bootstrap confidence interval for the indirect effect included zero (CI = [−0.051, 0.074]), suggesting that the mediation effect was not significant. However, under high levels of authentic leadership (Z = 5.029), the 95% confidence interval excluded zero (CI = [0.033, 0.176]), indicating a significant mediated effect. These results demonstrate that the indirect pathway ‘teachers’ well-being → perceived insider status → innovative work behavior’ varies significantly depending on the level of authentic leadership. In particular, high levels of authentic leadership strengthen the mediating role of perceived insider status. Therefore, Hypothesis 5 is supported.

## 5. Discussion

The findings of this study offer important theoretical and practical insights into the psychological and organizational mechanisms that shape teachers’ innovative work behavior. By integrating individual affective states with organizational context, it sheds light on how teacher well-being, perceived insider status, and authentic leadership interact to foster innovation in schools.

### 5.1. Interpretation of the Findings

#### 5.1.1. The Predictive Role of Teacher Well-Being in Innovative Work Behavior

Our study indicated that teacher well-being significantly predicts innovative work behavior, aligning with previous findings by Kundu and Roy (2016). Higher levels of well-being enable teachers to develop a deeper appreciation for the value and meaning of their educational work, thereby transforming intrinsic motivation into actual innovative practices. Recent research further supports this positive link. Lin and Liu (2025) showed that adaptability is positively related to innovative work behavior and negatively related to stress, underscoring how well-being related resources buffer teachers against the pressures of change while promoting innovation. Similarly, Zargar et al. (2025), in a study of university faculty in Turkey, found that work engagement enhances teachers’ innovative behavior.

While previous research has highlighted the link between workplace well-being and innovation among university teachers (Cao et al., 2020), this study contributes by adopting an integrative perspective that combines life satisfaction, psychological well-being, and workplace well-being into a multidimensional construct. By doing so, it provides more comprehensive evidence for the motivational role of well-being in promoting innovative behavior among teachers. Moreover, a recent systematic review of teacher well-being literature revealed that 74.55% of existing studies focus on their antecedents, such as individual traits, organizational features, and work environment, while relatively little attention has been paid to its outcomes (S. Zhang et al., 2024). This study responds directly to that gap by positioning teacher well-being as a key antecedent of innovation.

#### 5.1.2. The Mediating Role of Perceived Insider Status

The results suggest a potential mechanism through which teacher well-being fosters innovative work behavior, highlighting the mediating role of perceived insider status (PIS). In response to L. Huang’s (2013) call to explore workplace-based psychological mechanisms linking well-being and innovation, PIS is introduced as a key resource rooted in organizational context. This aligns with organizational research where insider perceptions mediate the influence of leadership, support, and team trust on employee outcomes (Khan et al., 2025). Parallel insights can be drawn from student studies demonstrating that school belonging predicts engagement, satisfaction, and adjustment (Urke et al., 2025; Medina et al., 2025). Extending this to educational contexts, the findings indicate that when teachers feel like valued insiders, they are more willing to invest in innovation. A strong perception of insider status helps translate positive emotional experiences into proactive behaviors by reinforcing emotional commitment and role-based engagement within the school.

The role of PIS identified here resonates with but also extends findings from other cultural contexts. In Western settings, the well-being–innovation relationship is often explained through motivational constructs such as self-efficacy, job engagement, or intrinsic motivation (Kwon & Kim, 2020). By contrast, our study highlights the psychosocial pathway of insider perceptions, which is particularly salient in collectivist cultural environments like China, where professional identity and relational belonging strongly influence work behavior (Zhai et al., 2025; Xing et al., 2025). This cross-cultural contrast suggests that while well-being universally supports innovation, the mechanisms may differ by context: motivational drivers may dominate in individualistic societies, whereas social-relational resources, such as insider status, are more critical in collectivist contexts.

PIS also enriches the theoretical foundation of teacher innovation research. Existing reviews highlight a lack of coherent theoretical frameworks, with limited application of theories such as self-efficacy or social cognitive theory (S. Liu et al., 2024). Drawing on broaden-and-build theory of positive emotions, this study frames teacher well-being not merely as a temporary emotional state, but as a psychological resource that broadens cognitive flexibility, builds emotional resilience, and strengthens organizational identification. This process enhances teachers’ sense of insider status, which in turn promotes teachers’ positive working behaviors like innovation. Thus, our research extends the relevance of the broaden-and-build theory in educational settings and suggests that fostering organizational belonging may be an effective strategy for promoting teacher innovation.

#### 5.1.3. The Moderating Role of Principal Authentic Leadership

The results further suggest that principal authentic leadership strengthens both the direct link between PIS and innovation and the indirect pathway from well-being through PIS. These findings contribute to a more nuanced understanding of how authentic leadership facilitates teacher innovation, while also extending the explanatory power of social information processing theory within educational contexts. Responding to calls for research on authentic leadership beyond predominantly Western contexts (Owusu-Bempah et al., 2014), this study provides evidence of its positive impact on teacher attitudes and behaviors within an Eastern cultural setting.

Prior studies have typically treated principal leadership as an antecedent of teachers’ innovative work behavior, focusing on the direct or indirect effects of authentic leadership (Shengnan & Hallinger, 2021). However, less attention has been paid to how authentic leadership may serve as a contextual enhancer within teachers’ psychological processes, particularly as a moderator in the pathway between individual cognitive states and behavioral outcomes (Ahmed, 2024). This study responds to and extends this line of inquiry by demonstrating that teachers’ sense of being organizational insiders and their willingness to engage in extra-role innovative behaviors are significantly shaped by their principal’s leadership.

According to social information processing theory, individuals tend to rely on external cues when navigating uncertain or complex work environments. As a stable and credible signal, authentic leadership conveys institutional trust, transparency, and value alignment. Such cues affirm teachers’ worth within the school, reinforcing their identity as insiders and creating a supportive psychological climate. This nuanced mechanism highlights that authentic leadership not only facilitates innovation through structural support but also by cultivating belonging and organizational commitment, which are particularly salient in collectivist cultural settings. In this context, teachers are more likely to transform positive emotional states into innovation-oriented motivation and action. By validating this pathway, the study not only expands the theoretical scope of authentic leadership as a situational moderator but also emphasizes that leadership shapes not just whether teachers innovate, but how they mobilize emotional and identity-based resources to do so.

### 5.2. Practical Implications

The findings of this study yield several valuable insights for school leadership and teacher professional development practices aimed at fostering innovation.

First, school leaders should place greater emphasis on enhancing teachers’ well-being as a means of stimulating their innovative work behavior. As knowledge-based professionals with strong intrinsic motivation, teachers often prioritize psychological fulfillment and professional satisfaction over material rewards. Well-being functions as a positive emotional state that promotes cognitive flexibility, openness to experience, and divergent thinking (P. Liu et al., 2023). These factors are essential for generating and implementing novel ideas in teaching. To support this, school leaders should integrate teacher well-being into routine managerial considerations by conducting regular assessments of teachers’ emotional and professional states, offering responsive feedback, and fostering a supportive culture that prioritizes teachers’ holistic development. Systematic efforts to cultivate well-being, such as workload management, offering meaningful recognition, and supporting professional autonomy, can activate teachers’ intrinsic motivation to innovate.

Second, our findings further revealed that approximately 31% of the effect of teacher well-being on innovative work behavior operates indirectly through perceived insider status. This proportion is noteworthy because it indicates that nearly one-third of the motivational benefits of teacher well-being are not directly translated into innovative outcomes, but rather depend on teachers’ sense of organizational belonging. This underscores the importance of cultivating an inclusive school climate that strengthens insider perceptions. Without such perceptions, even teachers with high well-being may find it difficult to transform their positive emotional resources into innovation-oriented behaviors. Principals should empower teachers through increased decision-making autonomy, recognition of innovative risk-taking, and meaningful involvement in policy discussions (Miller & Gkonou, 2025). Additionally, recognizing and celebrating teachers’ contributions during staff meetings or school-wide events reinforces their visibility and value within the organization. Principals can also strengthen relational trust by conducting informal classroom visits followed by constructive, appreciative feedback, rather than top-down evaluations. In the context of China’s ongoing education reforms, such as the ‘Double Reduction’ policy, principals can position teachers as co-designers of new after-school programs or assessment models, thereby affirming their professional expertise and insider status. As teachers internalize school goals and identify with the collective vision, they become more inclined to initiate and sustain innovative behaviors that benefit school development.

Finally, principals should embody authentic leadership qualities to create a psychologically safe and innovation-friendly school climate. The process of innovation often involves dealing with uncertainty, proposing unconventional ideas, and facing resistance. These challenges require strong institutional trust and support. Authentic leaders can foster this environment by demonstrating self-awareness and ethical integrity, becoming role models who inspire initiative. They should actively listen, integrate diverse perspectives in decision-making, and maintain transparent and inclusive communication with staff (L. Zhang et al., 2024). By cultivating openness and mutual respect, principals not only reinforce teachers’ organizational commitment but also empower them to experiment, collaborate, and take calculated risks in pursuit of pedagogical innovation. These leadership behaviors collectively contribute to a climate where innovation is not only permitted but encouraged and sustained.

## 6. Limitations

Several limitations should be noted that may suggest directions for future research. First, the sample was drawn exclusively from primary and secondary schools in City B. While the study collected a sufficient number of teacher responses, the geographical scope of the sampling remains limited, which may affect the applicability of the findings. As a result, the conclusions should be interpreted with caution when generalizing to schools in other regions with different cultural, economic, or policy environments. Future studies should include educators from diverse geographic and institutional contexts to improve representativeness and external validity. Second, although data were collected from nine schools, the nested structure of teachers within schools was not fully analyzed using multilevel methods due to the small number of schools. This analytical choice may overlook potential school-level influences, such as organizational climate, that interact with individual teacher factors. Given the organizational nature of schools, future research should apply multilevel modeling to better account for hierarchical data and examine cross-level influences. Third, all data were based on teacher self-reports, which may be influenced by social desirability and leniency biases. In particular, the measurement of authentic leadership relied on teachers’ perceptions, thereby capturing their subjective evaluations of principals’ behaviors. Incorporating multiple data sources like classroom observations, peer or principal ratings, and teaching portfolios, could provide more objective and comprehensive insights. Fourth, the cross-sectional design limits our ability to establish causality between variables. Longitudinal studies are needed to track changes in teacher well-being, organizational identity, and innovative behavior over time, thereby strengthening internal validity and supporting more robust theoretical development. Finally, while this study treated teacher innovative work behavior as a unified construct, future research could differentiate between incremental and radical innovations, allowing for a more nuanced understanding of how well-being and leadership dynamics shape different forms of innovation.

## 7. Conclusions

Based on the broaden-and-build theory of positive emotions and social information processing theory, this research developed and tested a moderated mediation model using data from 508 primary and secondary school teachers. Results show that teacher well-being positively predicts innovative work behavior, with perceived insider status mediating this relationship. Moreover, principal authentic leadership strengthens both the direct link between insider status and innovation and the indirect path from well-being to innovation through insider status. These findings deepen understanding of the workplace-based psychological mechanisms linking well-being to innovation, emphasizing the role of insider status. The conceptualization of authentic leadership is also extended by highlighting its function as a contextual enhancer of teacher motivation and behavior. Therefore, our study offers empirical and theoretical support for fostering teacher well-being and organizational identification through supportive leadership, providing actionable insights for school leaders and policymakers seeking to promote sustainable teaching innovation.

## Figures and Tables

**Table 1:** CFA results and coefficient alpha of the scales.

Scale	χ^2^/df	RMSEA	SRMR	CFI	NFI	Coefficient Alpha
TWBS	5.498	0.094	0.040	0.936	0.923	0.961
IWBS	5.515	0.094	0.022	0.976	0.971	0.973
PISS	4.093	0.078	0.023	0.995	0.994	0.860
ALQ	5.835	0.098	0.030	0.961	0.954	0.966

Note. TWBS = Teacher Well-Being Scale, IWBS = Innovative Work Behavior Scale, PISS = Perceived Insider Status Scale, ALQ = Authentic Leadership Questionnaire.

**Table 2:** Comparison of measurement models.

Model	χ^2^/df	RMSEA	RMR	CFI	NFI
Hypothesized model: TWB; IWB; PIS; AL	3.747	0.074	0.038	0.900	0.869
Model 1: TWB+ IWB + PIS + AL	12.914	0.153	0.126	0.566	0.547
Model 2: TWB + PIS + AL; IWB	9.392	0.129	0.148	0.694	0.671
Model 3: PIS + AL; TWB; IWB	4.653	0.085	0.053	0.867	0.837

Note. TWB = teachers’ well-being, IWB = teachers’ innovative work behavior, PIS = perceived insider status, AL = principal authentic leadership.

**Table 3:** Descriptive statistics and Pearson correlation coefficients for the study variables (*N* = 508).

	M	SD	1	2	3	4
1. Innovative work behavior	4.082	0.677	—			
2. Principal authentic leadership	4.507	0.522	0.535 **	—		
3. Perceived insider status	4.310	0.605	0.507 **	0.613 **	—	
4. Teachers’ well-being	5.752	0.745	0.537 **	0.411 **	0.580 **	—

Note. ** *p* < 0.01.

**Table 4:** Results of regression.

	IWB		PIS		IWB		IWB	
	Model 1	Model 2	Model 3	Model 4	Model 5	Model 6	Model 7	Model 8
Control variables								
Position	0.830 ** (3.131)	0.565 * (2.512)	0.422 (1.771)	0.166 (0.848)	0.830 ** (3.131)	0.594 * (2.581)	0.596 ** (2.747)	0.576 ** (2.676)
Class teacher	0.041 (0.644)	0.032 (0.596)	0.010 (0.176)	0.001 (0.030)	0.041 (0.644)	0.035 (0.643)	0.015 (0.290)	0.024 (0.473)
Gender	−0.122 (−1.513)	−0.162 * (−2.390)	0.022 (0.309)	−0.017 (−0.288)	−0.122 (−1.513)	−0.134 (−1.929)	−0.126 (−1.923)	−0.108 (−1.655)
Age	−0.005 (−0.653)	−0.007 (−0.967)	0.007 (0.914)	0.005 (0.901)	−0.005 (−0.653)	−0.009 (−1.286)	−0.008 (−1.183)	−0.008 (−1.209)
Teaching experience	0.004 (0.534)	0.004 (0.652)	−0.007 (−1.045)	−0.006 (−1.255)	0.004 (0.534)	0.007 (1.225)	0.007 (1.283)	0.007 (1.312)
Education	−0.038 (−0.401)	−0.041 (−0.522)	0.003 (0.041)	−0.000 (−0.002)	−0.038 (−0.401)	−0.040 (−0.487)	−0.052 (−0.677)	−0.068 (−0.898)
Main effect								
TWB		0.483 ** (14.180)		0.466 ** (15.712)				
PIS						0.560 ** (13.013)	0.309 ** (6.012)	0.309 ** (6.057)
Moderating effect								
AL							0.470 ** (7.941)	0.555 ** (8.579)
PIS × AL								0.243 ** (3.119)
R^2^	0.032	0.310	0.022	0.346	0.032	0.277	0.358	0.371
Adjusted R^2^	0.018	0.299	0.008	0.335	0.018	0.266	0.347	0.358
*F*	2.357 *	28.022 **	1.597	32.944 **	2.357 *	23.925 **	30.918 **	29.287 **

Note. TWB = teachers’ well-being, IWB = teachers’ innovative work behavior, PIS = perceived insider status, AL = principal authentic leadership. * *p* < 0.05, ** *p* < 0.01.

**Table 5:** Results of mediating effect.

	Total Effect	Indirect Effect	95% Boot CI	Direct Effect	Conclusion	Ratio
TWB=>PIS=>IWB	0.483 **	0.151	0.108~0.227	0.332 **	partial mediation	31.30%

Note. TWB = teachers’ well-being, IWB = teachers’ innovative work behavior, PIS = perceived insider status. ** *p* < 0.01.

**Table 6:** Test of moderated mediation.

Mediating Variable	AL	Effect	BootSE	Lower LCI	Upper LCI
PIS	M − 1 SD	0.012	0.032	−0.051	0.074
	M	0.056	0.030	−0.001	0.118
	M + 1 SD	0.100	0.037	0.033	0.176

Note. PIS = perceived insider status, AL = principal authentic leadership.

## Data Availability

The data that support the findings of this study are available from the corresponding author upon reasonable request.
